# Cerebrospinal fluid dopamine 3-O-sulfate as a novel biomarker for predicting motor complications in Parkinson’s disease: insights from the PPMI cohort

**DOI:** 10.1186/s12967-026-07761-7

**Published:** 2026-01-28

**Authors:** Jieshan Chi, Rui Yang, Piao Zhang, Siming Rong, Mengfei Cai, Yuhu Zhang

**Affiliations:** 1 Department of Neurology, Guangdong Neuroscience Institute, Guangdong Provincial People’s Hospital, Guangdong Academy of Medical Sciences, Southern Medical University, No. 106 Zhongshan Er Road, Guangzhou, Guangdong Province 510080 China; 2 Department of Emergency, Guangdong Provincial People’s Hospital, Guangdong Academy of Medical Sciences, Southern Medical University, Guangzhou, Guangdong Province 510080 China

**Keywords:** Parkinson’s disease, Levodopa, Motor complications, Dopamine 3-O-sulfate, Metabolomics

## Abstract

**Background:**

Long-term levodopa treatment for Parkinson’s disease (PD) is often complicated by motor fluctuations and dyskinesia. Predictive biomarkers for these debilitating side effects are currently lacking, hindering personalized treatment.

**Objectives:**

This study aimed to characterize the cerebrospinal fluid (CSF) metabolome across the PD continuum, distinguish disease-related from medication-related changes, and identify predictive biomarkers for levodopa-induced motor complications.

**Methods:**

We analyzed targeted CSF metabolomic data from the Parkinson’s Progression Markers Initiative (PPMI) cohort, which included healthy controls, prodromal, and PD participants. Statistical analyses revealed differentially abundant metabolites. The association of the metabolite dopamine 3-O-sulfate (DA3S) with motor complications was assessed using logistic regression and decision tree models.

**Results:**

DA3S was the most significantly altered metabolite in PD patients, with its elevation exclusively driven by levodopa treatment. DA3S levels were strongly correlated with levodopa exposure (LEDD) and demonstrated a significant independent association with the development of motor complications. A multivariable model combining DA3S, disease duration, and LEDD was used to predict motor complications, with an AUC of 0.806. A decision tree further confirmed the value of DA3S for risk stratification in specific patient subgroups.

**Conclusions:**

CSF DA3S is a pharmacodynamic marker of central levodopa metabolism and a robust, independent predictor of the onset of motor complications in PD patients. When combined with clinical variables, it facilitates effective risk stratification, providing a novel tool for personalizing therapy to mitigate treatment-related adverse effects.

**Graphical Abstract:**

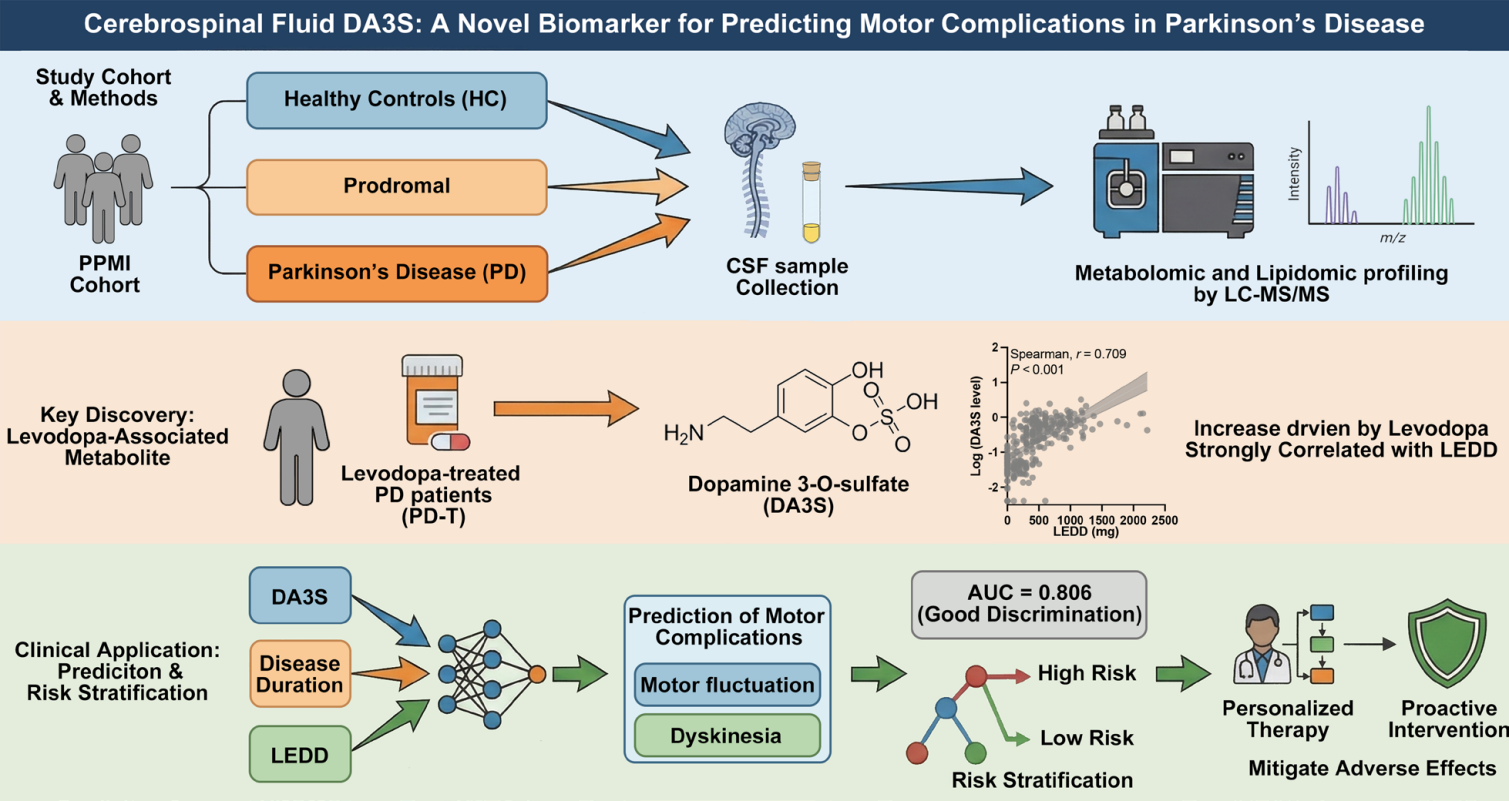

**Supplementary Information:**

The online version contains supplementary material available at 10.1186/s12967-026-07761-7.

## Highlights

CSF Dopamine 3-O-Sulfate (DA3S) is identified as a potent pharmacodynamic marker of central levodopa metabolism.

Elevated DA3S levels are independently associated with the risk of levodopa-induced motor complications in PD patients.

A multivariable model combining DA3S, LEDD, and disease duration demonstrates high predictive accuracy (AUC = 0.806).

DA3S provides a novel tool for risk stratification and personalized levodopa therapy management.

**Supplementary Information:**

The online version contains supplementary material available at 10.1186/s12967-026-07761-7.

## Introduction

Parkinson’s disease (PD) is a neurodegenerative disorder characterized by the progressive loss of dopaminergic neurons in the substantia nigra, leading to striatal dopamine deficiency and hallmark motor symptoms, including resting tremor, rigidity, and bradykinesia, as well as various nonmotor symptoms [1]. Although levodopa remains the gold-standard therapy for effectively replenishing dopamine and providing initial symptom relief, long-term use leads to motor complications—such as wearing-off [2] and levodopa-induced dyskinesia (LID) [3]—in approximately 50% of patients after five years [4]. The mechanisms underlying the development of these complications are complex and not fully understood but are thought to be related to pulsatile dopaminergic stimulation [5]. Motor complications severely impair patients’ functional mobility and quality of life while also generating increased health care costs and caregiving demands that place a substantial economic burden on families and society [6]. Currently, reliable biomarkers for predicting the risk of developing these motor complications are lacking, hindering the development of personalized treatment strategies.

Metabolomics provides a powerful tool for elucidating PD pathophysiology and identifying biomarkers. Plasma metabolomics has revealed alterations in amino acids, lipids, and phosphorylated metabolites in patients with PD, which may assist in distinguishing disease stages and identifying prodromal cases [7–9]. Notably, metabolic changes may precede clinical symptoms, as demonstrated in the UK Biobank cohort, where baseline metabolite levels were associated with subsequent PD development [10]. Additionally, metabolomics has been applied to evaluate the effects of pharmacological interventions, such as levodopa, on metabolic networks [11, 12], highlighting the need to distinguish drug-induced metabolic shifts from those inherent to the disease process [11].

In contrast to plasma metabolomics, cerebrospinal fluid (CSF) is in direct contact with brain tissue, and its metabolic composition more closely reflects biochemical alterations in the central nervous system. Changes in monoamine metabolite levels in the CSF have been directly linked to the pathological mechanisms of PD [13]. CSF metabolomics also offers greater sensitivity in detecting PD-related protein abnormalities, such as α-synuclein and its autoantibodies, whereas plasma metabolomics is often confounded by systemic metabolic influences [14, 15]. However, whether medication-associated CSF metabolites—such as dopamine 3-O-sulfate (DA3S)—could serve as predictive markers for motor complications, thereby augmenting clinical assessment, remains unexplored.

By utilizing the PPMI cohort, this study aimed to define the evolving CSF metabolomic landscape across the PD continuum—from health and prodromal stages to established disease—and to dissect the origin of these metabolic changes by distinguishing alterations inherent to the disease pathophysiology from those secondary to levodopa administration. We further systematically characterized the CSF metabolome to identify predictive biomarkers for levodopa-induced motor complications, focusing on DA3S, and to evaluate whether such medication-related metabolites can serve as biomarkers, alone or in combination with clinical variables, to predict the risk and severity of motor complications. This work seeks to provide a metabolic framework for understanding treatment consequences and to identify novel tools supporting precision medicine in PD management.

## Patients and methods

### Study population and design

This study utilized data from the Parkinson’s Progression Markers Initiative (PPMI) database (www.ppmi-info.org), a comprehensive observational international study designed to identify biomarkers for PD progression. All participants enrolled in the PPMI underwent comprehensive clinical phenotyping and biological characterization, including a series of standardized clinical assessments. Patients with PD were followed up annually with detailed documentation. In accordance with the predefined PPMI inclusion criteria, the participants were categorized into three groups: (1) the PD group, which consisted of individuals diagnosed with PD; and (2) the prodromal group, which included individuals in the prodromal stage of PD—a phase preceding the onset of characteristic motor symptoms such as tremor, rigidity, and bradykinesia. During this stage, patients may exhibit nonmotor symptoms or abnormalities in biomarkers, such as hyposmia, REM sleep behavior disorder, or constipation, as well as early dopaminergic system alterations observed on neuroimaging, while still maintaining largely normal motor function; and (3) the healthy control (HC) group, comprising individuals with no history of neurological disorders, no first-degree family history of PD, and normal dopaminergic imaging. Clinical characterization was performed using standardized instruments.

All participants were assessed with the Movement Disorder Society-Sponsored Revision of the Unified Parkinson’s Disease Rating Scale (MDS-UPDRS), the gold standard for evaluating PD severity. The MDS-UPDRS consists of four parts: Part I (Nonmotor Aspects of Experiences of Daily Living), Part II (Motor Aspects of Experiences of Daily Living), Part III (Motor Examination), and Part IV (Motor Complications). Global cognitive function was evaluated using the Montreal Cognitive Assessment (MoCA). Demographic information—including age, sex, and body mass index (BMI)—was also collected. CSF samples were obtained from participants, and only those with concurrent follow-up data at the time of CSF collection were included in the analysis. Participants lacking corresponding follow-up records were excluded. For PD patients, disease duration and medication history were documented. The levodopa equivalent daily dose (LEDD) was calculated for all medicated PD patients on the basis of the PPMI LEDD log.

Approval for the PPMI study protocol was obtained from the institutional review boards of all the participating sites. Written informed consent was obtained from all participants prior to their inclusion in the study. The secondary data analysis performed in this study adhered to the PPMI Data and Publications Committee (DPC) guidelines and was formally reviewed by the DPC.

### Metabolomic profiling

Targeted metabolomic and lipidomic profiling of CSF samples was performed using liquid chromatography coupled with tandem mass spectrometry (LC‒MS/MS). Samples were prepared using a standardized extraction protocol with stable isotope-labeled internal standards for quantification. Data were processed using MultiQuant and Skyline software, with peak areas normalized to internal standards and corrected for batch effects using ComBat. Analytes with missing values in more than 70% of the samples were excluded from the subsequent analysis.

### Statistical analysis

Baseline characteristics of the PD, prodromal, and HC groups were compared using appropriate statistical tests (ANOVA for continuous variables and chi-square tests for categorical variables). For the metabolomic data, the intergroup differences in metabolite levels were assessed using analysis of variance (ANOVA). Post hoc analysis for significant metabolites was performed using Tukey’s honest significant difference (HSD) test to control for multiple comparisons. The false discovery rate (FDR) method was applied to adjust p values for multiple testing across all measured metabolites. The associations between continuous variables and clinical scores were evaluated using Spearman’s correlation analysis. To investigate the relationships between risk factors and the presence of motor complications (defined as an MDS-UPDRS Part IV score > 0), univariable and multivariable logistic regression models were employed. DA3S values were log10-transformed prior to regression analysis to improve the data distribution. The discriminative ability of the models was evaluated by calculating the areas under the receiver operating characteristic curves (AUCs). Cutoff values were derived on the basis of Youden’s index to maximize model sensitivity and specificity. Model performance was further assessed by determining accuracy, sensitivity, specificity, and predictive values at the optimal probability threshold. Model robustness was evaluated using internal bootstrap validation. Multicollinearity among the predictors in the multivariable model was checked using the variance inflation factor (VIF). A decision tree model was constructed to provide clinically interpretable cutoff values for predicting motor complications.

All the statistical analyses were performed using R software (version 4.5.1; R Foundation for Statistical Computing, Vienna, Austria). A two-tailed p value < 0.05 was considered to indicate statistical significance.

## Results

### Study population and baseline clinical assessments

Among the 597 participants enrolled and analyzed in this study, 279 were patients with PD, 226 were prodromal individuals, and 92 were healthy controls. A flowchart of the participant screening process is shown in Figure S1. Table 1 presents the baseline characteristics of the study participants. As shown, no statistically significant differences were observed among the three groups in terms of age, sex distribution, or BMI, indicating good comparability across groups at baseline. Significant differences were observed in the assessments of neuropsychological and motor functions. The MoCA scores differed significantly overall, with post hoc tests confirming lower scores in the PD group than in the HC group; however, the scores in the prodromal group did not differ significantly from those in either the PD or HC groups. Consistent with the established literature, a significant between-group difference was observed in the scores for the MDS-UPDRS Part I, which assesses nonmotor aspects of daily living. Compared with the HC group, the prodromal group already exhibited significantly aggravated nonmotor symptoms, with a 52% increase in scores. As expected, the PD group scored significantly higher than both the prodromal and the HC groups did. With respect to motor symptoms, the PD group demonstrated significantly higher scores than both the prodromal group and the HC group on both the MDS-UPDRS Part II (Motor Experiences of Daily Living) and Part III (Motor Examination). No significant difference was observed between the prodromal and HC groups on these motor scales.

**Table 1:** Baseline demographic and clinical characteristics of study participants

^Characteristic^	PD (*n*=279)	Prodromal (*n*=226)	HC (*n*=92)	ANOVA (*p*−value)	Post−hoc (*p*−value)		
					PD vs. HC	PD vs. Prodromal	Prodromal vs. HC
Age, years(mean±sd)	63.5 ± 8.7	63.9 ± 7.3	63.5 ± 9.9	0.869			
Female, n (%)	121 (43.4%)	102 (45.1%)	41 (44.6%)	0.922			
BMI (mean±sd)	26.74 ± 4.44	27.66 ± 4.95	26.83 ± 5.15	0.083			
MoCA score (mean±sd)	26.47 ± 2.87	26.75 ± 2.43	27.42 ± 1.91	0.0095*	0.0066*	0.4647	0.0868
MDS−UPDRS Part I score (mean±sd)	7.79 ± 5.46	5.20 ± 4.18	3.43 ± 3.17	<0.001*	<0.001*	<0.001*	0.007*
MDS−UPDRS Part II score (mean±sd)	7.35 ± 5.21	1.12 ± 2.13	0.64 ± 1.29	<0.001*	<0.001*	<0.001*	0.577
MDS−UPDRS Part III score (mean±sd)	^24.57 ± 11.54^	2.71 ± 3.31	1.25 ± 2.20	<0.0001*	<0.001*	<0.001*	0.2879

Comparison of age, sex, BMI, MoCA scores, and MDS-UPDRS scores across Parkinson’s disease (PD), Prodromal, and Healthy Control (HC) groups. Data are presented as mean ± standard deviation for continuous variables and n (%) for categorical variables. P-values from ANOVA or Chi-square tests are shown, along with post-hoc pairwise comparison p-values

### Identification of CSF DA3S as a levodopa-associated metabolite

**Fig. 1:**
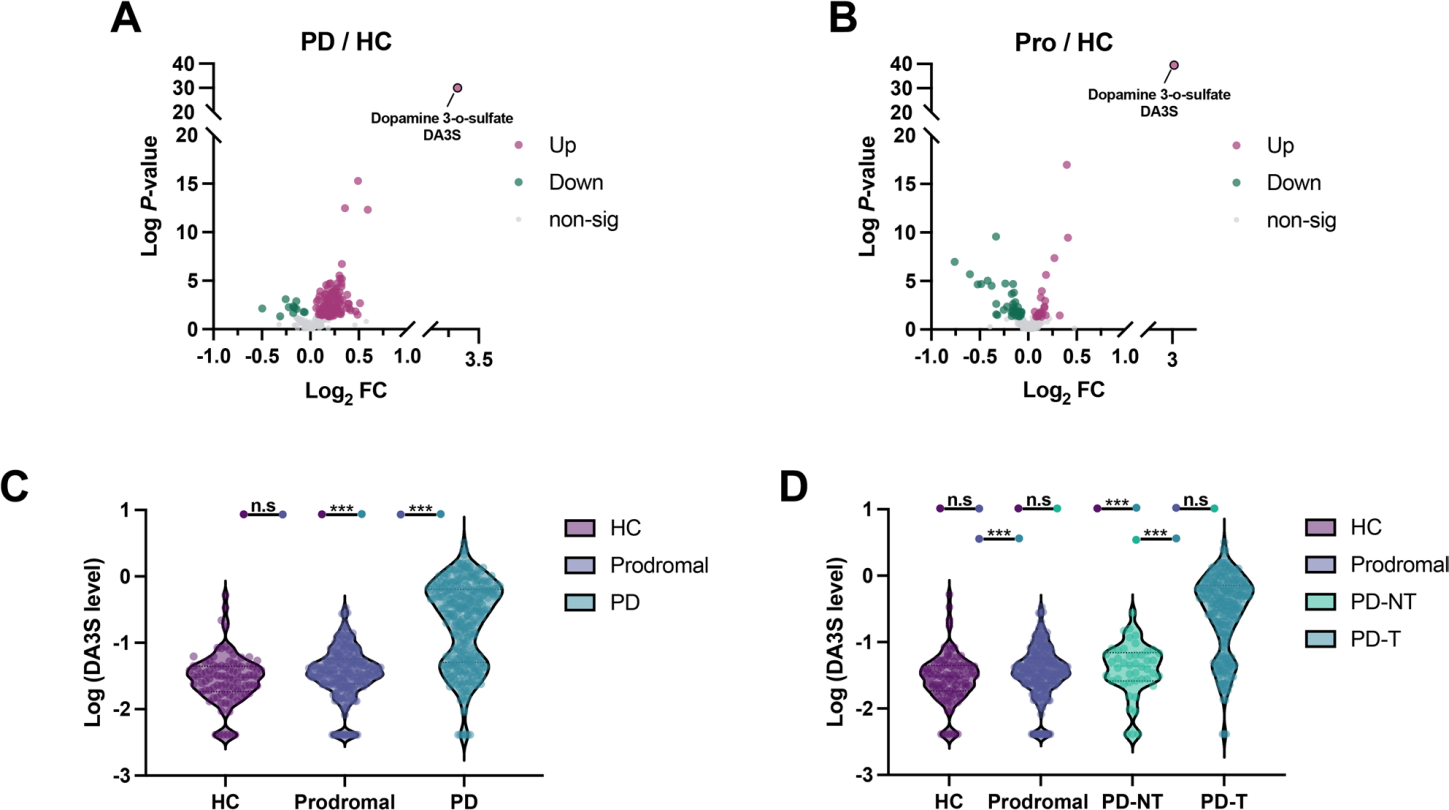
CSF DA3S levels were markedly elevated in patients with levodopa-treated Parkinson’s disease. (**A**–**B**) Volcano plot identifying DA3S as the most significantly altered metabolite in PD. (**C**) DA3S levels were significantly higher in the PD group than in the prodromal and HC groups. (**D**) DA3S elevation was specific to levodopa-treated PD patients (PD-T) compared with the prodromal, HC, and untreated PD (PD-NT) groups

On the basis of metabolomic data from the PPMI cohort, this study compared CSF metabolite levels among the three groups and identified 93 metabolites with significant intergroup differences (Table S1). Widespread alterations were observed in the polyamine metabolism pathway (including that of cadaverine, N-acetylputrescine, N1-acetylspermidine, and putrescine, among others), as well as in molecules related to catecholamine and purine metabolism, suggesting their potential involvement in the pathophysiology of PD. Notably, DA3S exhibited the most pronounced difference across groups (F value = 101.63; FDR-adjusted q value = 1.18 * 10^− 36^), with a level of statistical significance far exceeding that of all the other measured metabolites (Fig. 1A-B). Post hoc analysis further revealed that the DA3S levels in the PD group significantly differed from those in both the prodromal group and the HC group, whereas no significant difference was detected between the prodromal group and the HC group (Fig. 1C).

However, as DA3S is a major sulfated conjugate metabolite of dopamine, whether its concentration in CSF is influenced by levodopa medication remains unclear. To address this question, we divided PD patients into two subgroups: a PD-T group comprising those treated with levodopa or related medications and a PD-NT group consisting of untreated patients. The results showed that the PD group had the highest mean DA3S level (0.502), which was significantly different from that of all the other groups (*p* < 0.001). In contrast, the PD-NT, prodromal, and HC groups exhibited similar DA3S levels, with means ranging from 0.044 to 0.055 and no significant differences among them (Fig. 1D). These findings demonstrate conclusively that the marked increase in DA3S observed in the PD group is associated with levodopa administration.

## Clinical utility of the DA3S in predicting and stratifying motor complications

**Fig. 2:**
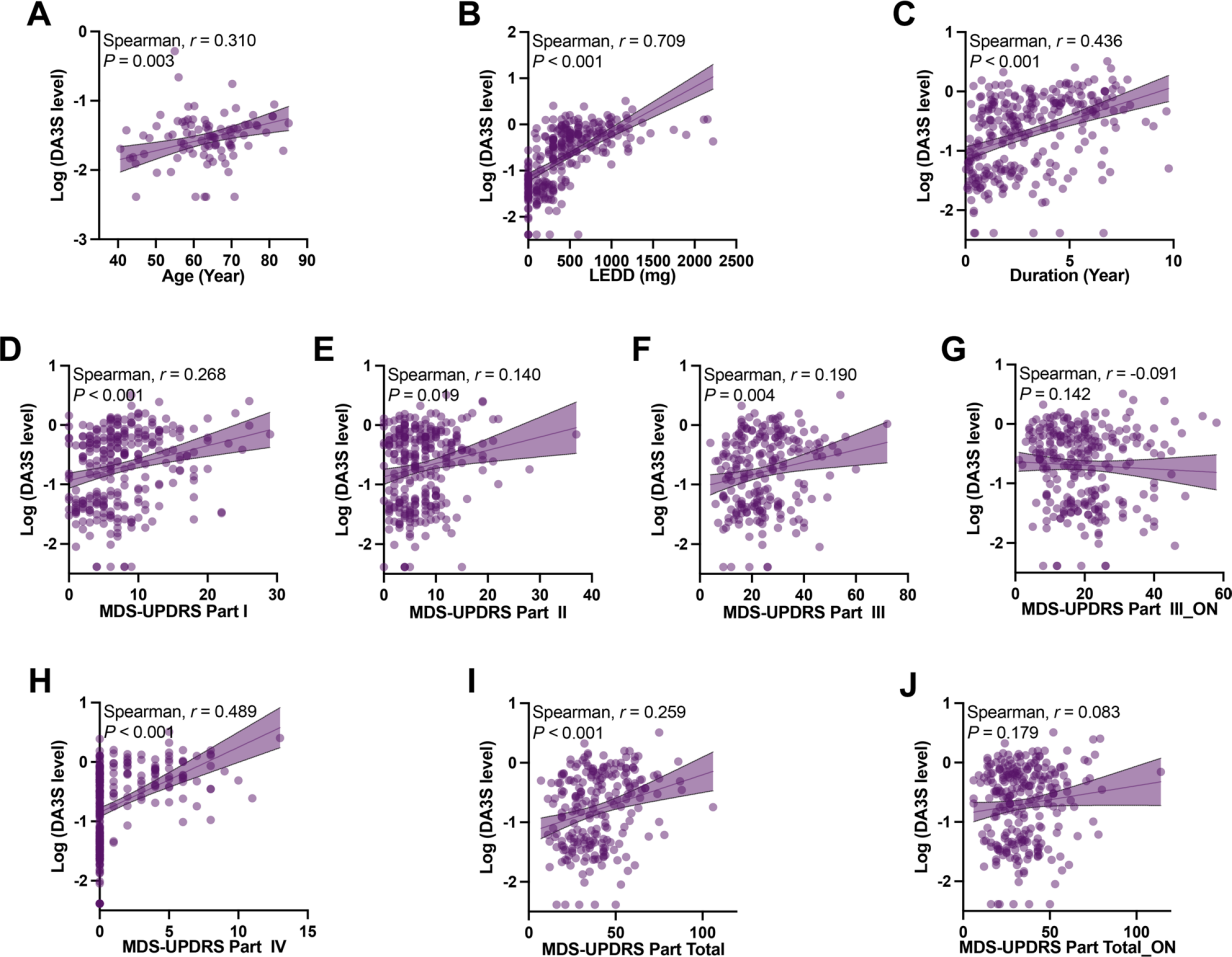
Correlations between CSF DA3S level and clinical parameters. DA3S level was (**A**) weakly correlated with age in HCs and strongly positively correlated with (**B**) LEDD and (**C**) disease duration in PD patients. (**D**–**J**) Correlation between the DA3S level and MDS-UPDRS score in PD patients

We first assessed whether DA3S accumulates with age by analyzing its correlation in healthy controls and observed only a weak positive correlation (*r* = 0.2), suggesting a slight increase with age (Fig. 2A). To evaluate the role of DA3S in PD, we examined its relationship with key clinical measures, including MDS-UPDRS subscores, LEDD, and disease duration. DA3S was strongly positively correlated with LEDD (*r* = 0.7091; Fig. 2B), supporting its role as a downstream metabolite of levodopa exposure. A moderate correlation was also observed with disease duration (*r* = 0.436; Fig. 2C), which is consistent with cumulative medication effects. Correlation analyses between the DA3S and MDS-UPDRS subscores (Fig. 2D–J) revealed a notable positive association with Part IV (*r* = 0.489), indicating a link to motor complications—a key challenge in PD management. This finding is of particular clinical relevance, given the central role of motor complications in PD management. It should be noted, however, that the zero-inflated distribution of Part IV data warrants specialized statistical methods for accurate interpretation.

**Fig. 3:**
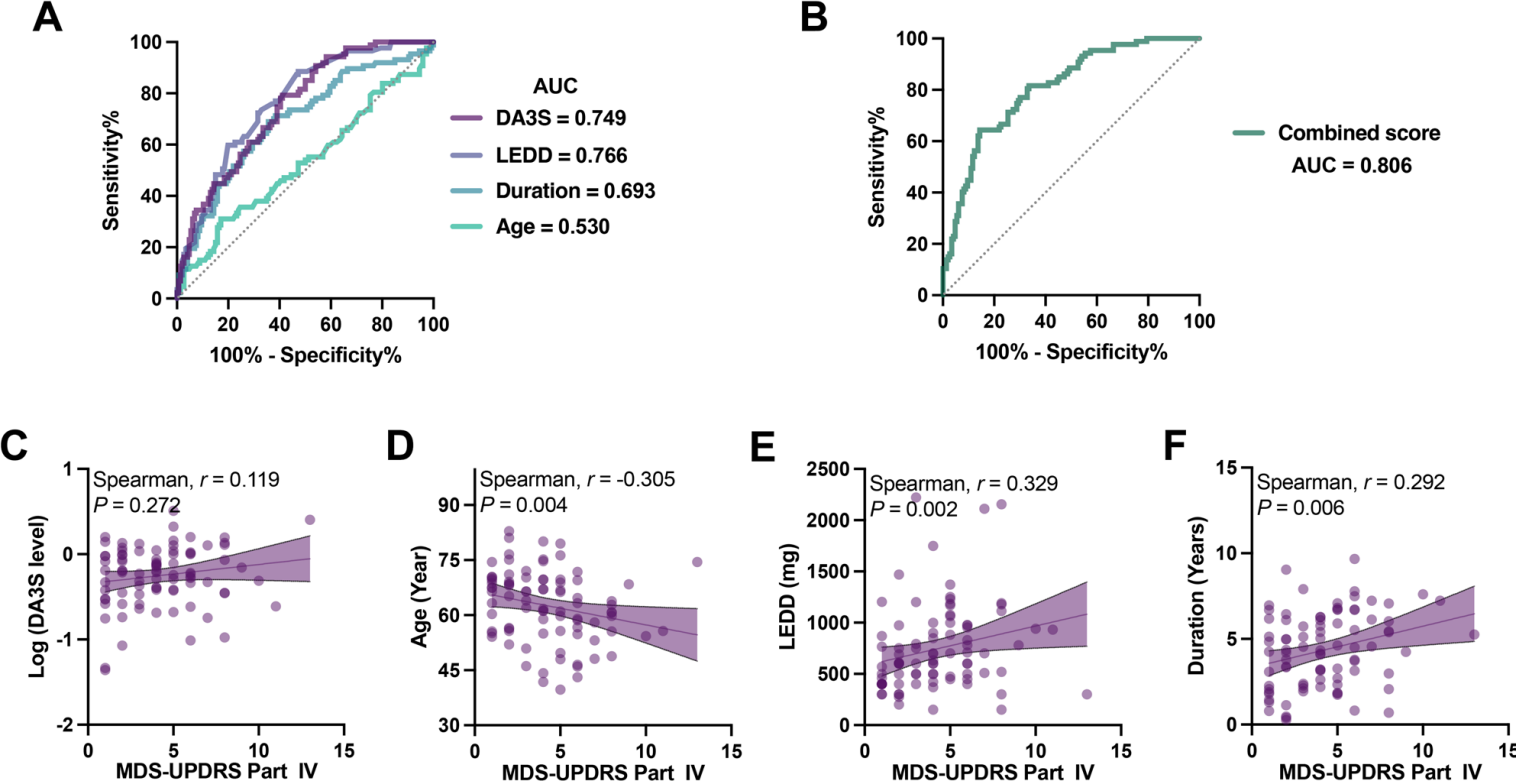
Predictive performance for motor complications. (**A**) ROC curves of univariable models incorporating DA3S, LEDD, disease duration, and age. (**B**) ROC curve of the multivariable model combining DA3S, LEDD, and disease duration. (**C**–**F**) Factors associated with the severity of established motor complications

The MDS-UPDRS-Part IV assesses motor fluctuations and dyskinesias related to long-term levodopa treatment. We defined motor complication status on the basis of this subscale, with a score > 0 indicating presence. Univariable logistic regression was used to evaluate the associations of log10-transformed DA3S, LEDD, disease duration, and age with motor complications (Table 2). Each tenfold increase in the DA3S concentration increased the odds of motor complications by 8.25-fold (OR = 8.251; 95% CI: 4.181–18.053; *P* < 0.0001), indicating that it is a strong risk factor. Every 100 mg increase in LEDD increased the risk by approximately 34.9% (OR ≈ 1.349 per 100 mg), and each additional year of disease duration increased the risk by 40.4% (OR = 1.404). Age was not significantly associated (*P* = 0.328). With respect to the discriminative ability of the model, the predictive power varied among the factors. The area under the curve (AUC) values for the models containing DA3S, LEDD, and disease duration were 0.749, 0.766, and 0.693, respectively, indicating a certain degree of predictive value for motor complications (Fig. 3A). Age had an AUC of 0.530, which is consistent with its nonsignificant statistical result.

**Table 2:** Univariable and multivariable logistic regression analyses for predictors of motor complications

Variable	Single-factor logistic regression		Multivariate logistic regression	
	OR (95%CI)	P_value	OR (95%CI)	P_value
DA3S	8.251 (4.181–18.053)	< 0.0001	4.21 (1.994–9.733)	0.000355
LEDD	1.0031(1.002–1.004)	< 0.0001	1.001 (1-1.002)	0.006081
Duration	1.404 (1.228–1.619)	< 0.0001	1.251 (1.077–1.46)	0.003785
Age	0.985 (0.995–1.015)	0.3282		

*Odds ratios (OR) with 95% confidence intervals (95% CI) and p-values for the association of log10-transformed DA3S, Levodopa equivalent daily dose (LEDD), disease duration, and age with the presence of motor complications. The multivariable model includes DA3S, LEDD, and disease duration as independent predictors*

A multivariable logistic regression model incorporating DA3S, LEDD, and disease duration identified all three variables as independent predictors of motor complications (Table 2). Each log-unit increase in DA3S was associated with a fourfold increase in complication odds (OR = 4.21, *P* < 0.001), confirming its role as a potent biomarker. A longer disease duration (OR = 1.25 per year) and higher LEDD also remained significant independent risk factors. The model demonstrated good discrimination (AUC = 0.806; Fig. 3B). Using a probability threshold of 0.507 determined by maximizing Youden’s index (J = 0.488), the classification performance showed 77.3% accuracy, 63.2% sensitivity, 85.6% specificity, 72.4% positive predictive value, and 79.6% negative predictive value. All variance inflation factors were less than 1.5, indicating no substantial multicollinearity. The model demonstrated robust performance through internal validation. Bootstrap validation with 1000 iterations yielded an AUC of 0.810. Repeated split-sample validation further confirmed generalizability, with a median training AUC of 0.812 and a median test AUC of 0.797 (Fig. S2A). Calibration results showed a Brier score of 0.153, a calibration slope of 1.42, and an intercept of 0.01 (Fig. S2B). These results collectively support the model’s discrimination ability and predictive consistency.

On the basis of the results of the multivariate logistic regression analysis, we developed a decision tree algorithm for the risk stratification of motor complications. DA3S demonstrated strong risk-discriminatory value within specific subgroups. Among patients with LEDD ≥ 360 mg and disease duration < 3.14 years (*n* = 62), DA3S was the primary splitting variable at a cutoff of 0.209: patients with a DA3S < 0.209 (*n* = 12) had a 13.2% complication risk, whereas those with a DA3S ≥ 0.209 (*n* = 50) had a 54.9% risk—a 4.2-fold difference. Furthermore, among patients with a DA3S ≥ 0.209, a finer stratification based on LEDD revealed a clinically noteworthy “dose–risk paradox”: patients with an LEDD of 775–1032 mg had a lower risk (15.7%) than those with an LEDD < 775 mg (56.6%). These findings reveal the complex interaction between medication dosage and biomarker levels in determining complication risk. These results not only confirm the clinical value of DA3S as an important biomarker but also provide a scientific basis for developing individualized treatment strategies, offering a practical tool for the early identification of high-risk patients.

Among patients with motor complications, the DA3S level was not correlated with severity (Fig. 3C), suggesting that it does not influence intensity once complications occur. In contrast, younger age was associated with greater severity (Fig. 3D), whereas longer disease duration and higher cumulative dopaminergic exposure emerged as the principal determinants of worse outcomes (Fig. 3E–F). These findings indicate that complication severity reflects underlying disease progression and total medication burden rather than DA3S levels or age.

## Discussion

Levodopa crosses the blood–brain barrier and is predominantly decarboxylated to dopamine within neuronal cells by the enzyme aromatic L-amino acid decarboxylase (AADC) [16]. Dopamine is subsequently metabolized primarily by monoamine oxidase (MAO) and catechol-O-methyltransferase (COMT) to end products such as 3,4-dihydroxyphenylacetic acid (DOPAC) and homovanillic acid [17]. Notably, dopamine can also be involved in alternative metabolic pathways with potential clinical significance. Oxidative metabolism can generate reactive toxic species such as dopamine quinones and 3,4-dihydroxyphenylacetaldehyde, which are involved in disease progression and neurodegeneration [18]. Alternatively, sulfotransferases can sulfate dopamine at the 3-hydroxy group, forming DA3S [19]. DA3S is a sulfated derivative of dopamine and represents one of the activated molecules within the neuromodulatory system that undergoes sulfation modification [20, 21]. Studies have shown that DA3S levels are significantly reduced in patients with AADC deficiency, whereas in non-AADC patients receiving levodopa therapy, the levels of downstream metabolites—including DA3S—remain normal or elevated [22]. This finding indicates that its level may reflect the metabolic status of levodopa. Recent studies in rodent models suggest that dopamine sulfation can modulate aggregative behavior by enhancing free dopamine-mediated behavioral responses [20]. These findings suggest that sulfation plays a broader neuromodulatory role in the neural regulation of animal social behaviors and holds functional significance in the regulation of dopamine signaling. Compared with free dopamine, sulfated dopamine has distinct properties. The introduction of a sulfonate group significantly increases its water solubility, facilitating renal excretion [23, 24]. Concurrently, it is no longer metabolized by MAO or COMT and exhibits considerable stability within the body [25, 26]. Previous nontargeted serum metabolomic analysis revealed that the level of DA3S was significantly elevated in the serum of PD patients, with a fold change of 13.85 and a highly significant FDR, indicating that it is among the most markedly altered metabolites in patients with PD [27]. Similarly, our study from the PPMI cohort unequivocally identified DA3S as the metabolite with the most pronounced intergroup differences, significantly greater than all the other measured metabolites. This striking separation across diagnostic categories strongly suggests the potential utility of DA3S as a biochemical marker in PD.

Over time, multiple extrastriatal brain regions exhibit impaired regulation of dopamine production derived from levodopa [28], leading to excessive accumulation of dopamine or its precursors. In animal models of LID, the levels of levodopa and its metabolite 3-O-methyldopa are significantly elevated across all brain regions, particularly in extrastriatal areas [28]. This suggests that a fundamental issue in LID is the systemic dysregulation of levodopa metabolism rather than merely fluctuating dopamine levels confined to the striatum. Despite markedly high cerebral levodopa concentrations, striatal dopamine levels do not increase substantially. Instead, levodopa is extensively converted to dopamine in extrastriatal regions, where their levels are strongly correlated. This excess dopamine is preferentially metabolized by COMT to 3-methoxytyramine over MAO-mediated degradation to DOPAC, indicating a shifted metabolic pathway [28]. Chronic levodopa treatment also perturbs other systems, such as those involved in polyamine metabolism and endocannabinoid signaling, which may contribute to dyskinesia [11]. Abnormal levodopa metabolism disrupts neuronal activity in the striatum, representing a core mechanism underlying LID in PD patients following long-term levodopa therapy. The striatal dysregulation underlying LID may originate from the widespread brain-wide conversion of levodopa to dopamine, which alters neuronal signaling [28, 29]. Dyskinesia is linked to aberrant activity in specific regions, such as the external globus pallidus and subthalamic region, where neuronal overactivity is both a feature and a therapeutic target of dyskinesia [30]. Concurrently, levodopa metabolism generates reactive oxygen species and oxidized metabolites that induce mitochondrial/lysosomal dysfunction, exacerbating neuronal damage [16, 31]. Furthermore, dopamine–adenosine receptor antagonism disrupts striatal projection neuron activity [32].

These findings demonstrate that long-term levodopa treatment causes dysregulated dopamine metabolism across brain regions. Significant alterations in its metabolic patterns and byproducts are closely associated with the development of motor complications. As evidenced by our analysis, the elevated DA3S levels in PD patients are medication-driven, a conclusion supported by the comparable DA3S levels among drug-naïve PD patients, prodromal patients, and healthy controls. Thus, DA3S serves not as a diagnostic biomarker but as a pharmacodynamic marker of levodopa exposure.

Our findings indicate that DA3S not only reflects central levodopa dysregulation but also predicts motor complications, highlighting its clinical relevance and the critical objective of developing biomarkers for early identification. While objective tools—including digital motor assessments [33], vocal biomarkers [34], and machine learning-derived composite scores [35]—can monitor complications and treatment efficacy, a critical gap remains in early molecular biomarkers for predicting the onset of these complications or tracking treatment response [36]. In response to this gap, research is actively exploring the levels of dopamine metabolites in the CSF and plasma as potential indicators of dopaminergic function [37]. In our study, DA3S was independently associated with motor complications after adjusting for LEDD and disease duration, with the model showing strong discrimination and high specificity for identifying at-risk patients. As a potential predictor of motor complications, DA3S could effectively assist clinicians in anticipating disease progression, enabling timely treatment adjustments. This would allow for more precise titration of levodopa dosage and dosing intervals, thereby helping to prevent symptom exacerbation or worsening drug side effects resulting from delayed intervention [5, 38]. Such stratification would facilitate preemptive measures, such as the early introduction of adjunct therapies such as dopamine agonists or MAO-B inhibitors [39, 40]. For patients undergoing subthalamic nucleus deep brain stimulation (STN-DBS), the ability to predict motor complications could guide the optimization of stimulation parameters. Long-term follow-up studies have indicated that STN-DBS continues to ameliorate motor complications for more than 15 years [6], and the development of a real-time predictive capability may further enhance its therapeutic efficacy [41, 42].

Given the collective evidence, we hypothesize that DA3S accumulation represents a stable biochemical fingerprint of a critical pathological process induced by long-term levodopa therapy: the progressive loss of dopaminergic buffering capacity and the subsequent emergence of nonphysiological dopamine fluctuations. Therefore, elevated DA3S levels, which serve as an integrator of this process, are mechanistically linked to the development of motor complications, extending beyond a simple correlation with the duration or dosage of drug exposure. Whether DA3S has active biological functions or acts merely as an inert marker remains to be elucidated by future experimental studies.

However, among patients who developed such complications, younger age at onset was associated with greater symptom severity—a finding that partially diverges from previous reports focusing specifically on LID risk in younger patients [43]. This discrepancy may reflect challenges in the early recognition of motor complications. Early levodopa-related complications such as wearing-off phenomena are frequently misinterpreted as natural disease progression [44], while escalating levodopa requirements in advanced disease may exacerbate complications, creating a treatment-resistant cycle [45]. Early detection of levodopa-related complications remains critical for therapeutic optimization, particularly given the decreasing efficacy and increasing complication burden associated with long-term levodopa use [46, 47]. Early prediction of motor complications can reduce reliance on subjective assessment, overcome the limitations of patient self-reports, and guide more precise medication adjustments. This approach enhances the personalization of therapy, aiming to maintain levodopa doses above the therapeutic threshold for PD control while avoiding levels that induce motor complications, thereby ultimately preserving quality of life.

Notably, DA3S strongly predicted the development of motor complications but not their subsequent severity, suggesting distinct pathophysiological mechanisms for initiation versus progression. Complication severity correlated more closely with disease duration and cumulative LEDD than the DA3S level did. While the neurobiological consequences of DA3S accumulation require further elucidation, its robust association with complications independent of traditional risk factors supports its value as a novel biomarker. Dopamine sulfation may alter stability or receptor interactions, and behavioral studies suggest that sulfated dopamine may function as an independent signaling molecule [20]. Nevertheless, key questions persist regarding whether DA3S accumulation reflects specific metabolic pathway loading, possesses novel biological activities, or simply indicates dopamine metabolic flux overload. These possibilities require further investigation to elucidate the exact pathophysiological role of DA3S.

Several limitations warrant consideration. The cross-sectional nature of our analysis precludes definitive causal inferences regarding DA3S and the development of motor complications. While DA3S serves as a robust statistical predictor, longitudinal studies are needed to validate its prospective value and to determine whether early elevations in DA3S consistently precede the clinical onset of dyskinesia or fluctuations. Although the lack of an independent external validation cohort is a constraint due to the scarcity of paired CSF samples, we mitigated this by performing the strict internal validation, including Bootstrap validation and split-sample internal validation, which confirmed the model’s robustness on unseen data. External validation in diverse populations is necessary to confirm their robustness. The mechanisms through which DA3S might contribute to disease progression or complication pathogenesis also require experimental investigation. Furthermore, the clinical translation of CSF DA3S measurement warrants practical consideration. CSF collection requires lumbar puncture, an invasive procedure justified only by clear clinical benefit. Therefore, future studies should investigate the correlation of DA3S levels in more accessible matrices, such as plasma or urine, to explore less invasive alternatives. Ultimately, the development of cost-effective, standardized assay kits will be crucial for enabling routine clinical implementation. In line with prior evidence, we note that potential confounders such as diet [48] and concomitant medications (e.g., MAO-B inhibitors, which may redirect dopamine metabolism toward sulfation) could influence DA3S levels. Future studies should therefore control for dietary factors and standardize the timing of medication intake relative to sample collection.

## Conclusion

In conclusion, our findings establish DA3S as a significant pharmacodynamic marker that strongly predicts the development of motor complications in PD patients. Its accumulation reflects both levodopa dosing and treatment duration, and it demonstrates independent predictive value beyond conventional risk factors. These results position DA3S as a promising biomarker, whose measurement in CSF could help identify patients at elevated risk for treatment-related complications and guide neurologists in optimizing levodopa dosing and treatment strategies, enabling proactive interventions to delay or mitigate motor complications.

## Supplementary Information

Below is the link to the electronic supplementary material.

Supplementary Material 1

## Data Availability

The data used in the preparation of this article were obtained from the Parkinson’s Progression Markers Initiative (PPMI) database (www.ppmi-info.org/access-data-specimens/download-data). For up-to-date information on the study, visit www.ppmi-info.org.

## Abbreviation

AADC: Aromatic L-amino acid decarboxylase
ANOVA: Analysis of variance
AUC: Area under the curve
BMI: Body mass index
CI: Confidence interval
COMT: Catechol-O-methyltransferase
CSF: Cerebrospinal fluid
DA3S: Dopamine 3-O-sulfate
DBS: Deep brain stimulation
DOPAC: 3,4-Dihydroxyphenylacetic acid
FDR: False discovery rate
HC: Healthy controls
HVA: Homovanillic acid
LC‒MS/MS: Liquid chromatography–tandem mass spectrometry
LEDD: Levodopa equivalent daily dose
LID: Levodopa-induced dyskinesia
MAO: Monoamine oxidase
MDS-UPDRS: Movement Disorder Society-Unified Parkinson's Disease Rating Scale
MoCA: Montreal Cognitive Assessment
OR: Odds ratio
PD: Parkinson's disease
PPMI: Parkinson's Progression Markers Initiative
ROS: Reactive oxygen species
STN-DBS: Subthalamic nucleus deep brain stimulation
SULT: Sulfotransferase
VIF: Variance inflation factor
