# Supplementary material for: Cerebrospinal fluid dopamine 3-O-sulfate as a novel biomarker for predicting motor complications in Parkinson’s disease: insights from the PPMI cohort
Source: J Transl Med. 2026 Jan 28;24:178. doi: 10.1186/s12967-026-07761-7 (PMC12895786; doi:10.1186/s12967-026-07761-7)
Supplement: Supplementary file 1 — Supplementary Material 1 [file 12967_2026_7761_MOESM1_ESM.docx]

### Supplementary Material

**Cerebrospinal Fluid Dopamine 3-O-Sulfate as a Novel Biomarker for Predicting Motor Complications in Parkinson’s Disease: Insights from the PPMI Cohort**

Jieshan Chi^1,2^, Rui Yang^1^, Piao Zhang^1^, Siming Rong^1^, Mengfei Cai^1^, Yuhu Zhang^1*^

^1^Department of Neurology, Guangdong Neuroscience Institute, Guangdong Provincial People's Hospital, Guangdong Academy of Medical Sciences, Southern Medical University, Guangzhou, Guangdong Province, 510080, China.

^2^Department of Emergency, Guangdong Provincial People's Hospital, Guangdong Academy of Medical Sciences, Southern Medical University, Guangzhou, Guangdong Province, 510080, China.

*Correspondence to: Yuhu Zhang. Department of Neurology, Guangdong Neuroscience Institute, Guangdong Provincial People's Hospital (Guangdong Academy of Medical Sciences), Southern Medical University, No. 106 Zhongshan Er Road, Guangzhou, 510080, China. Email: zhangyuhu@gdph.org.cn.


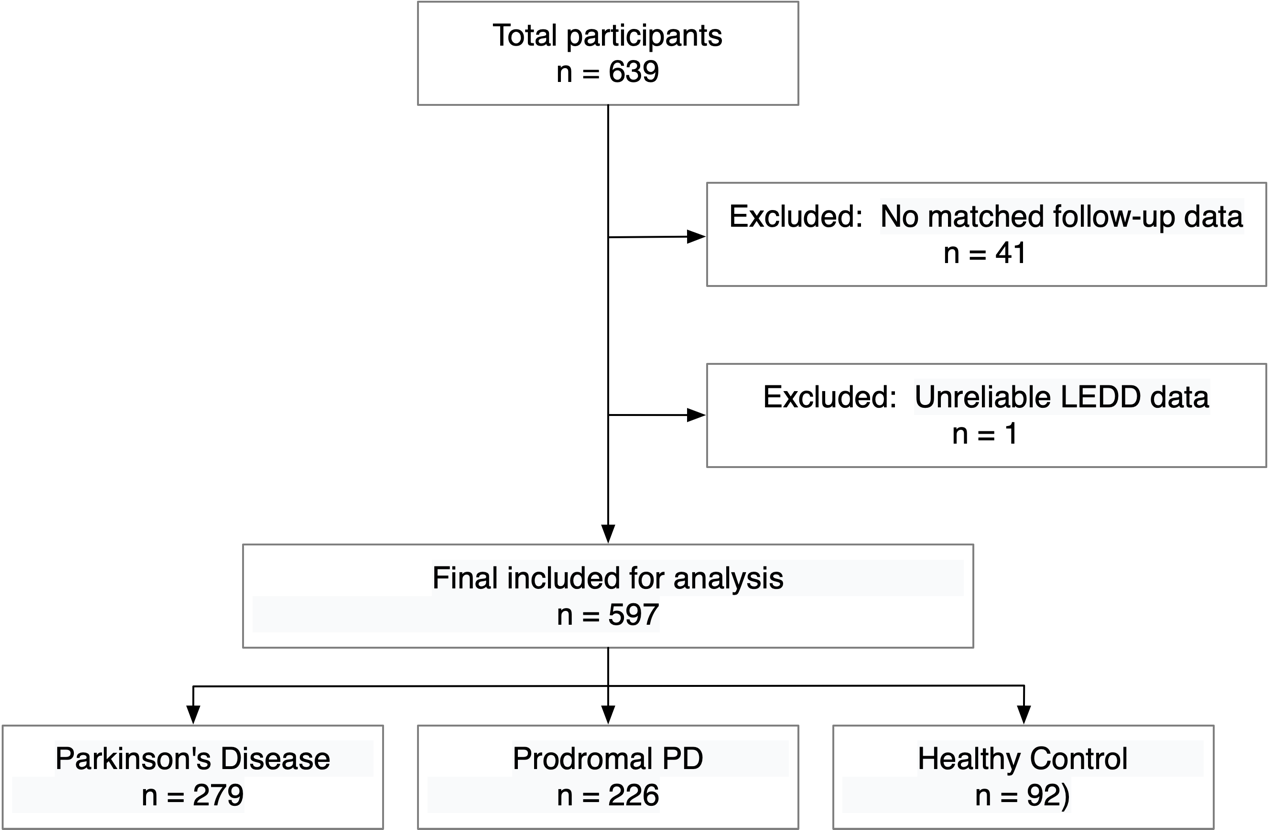


**Fig. S1** Participant screening and inclusion flow diagram.

**
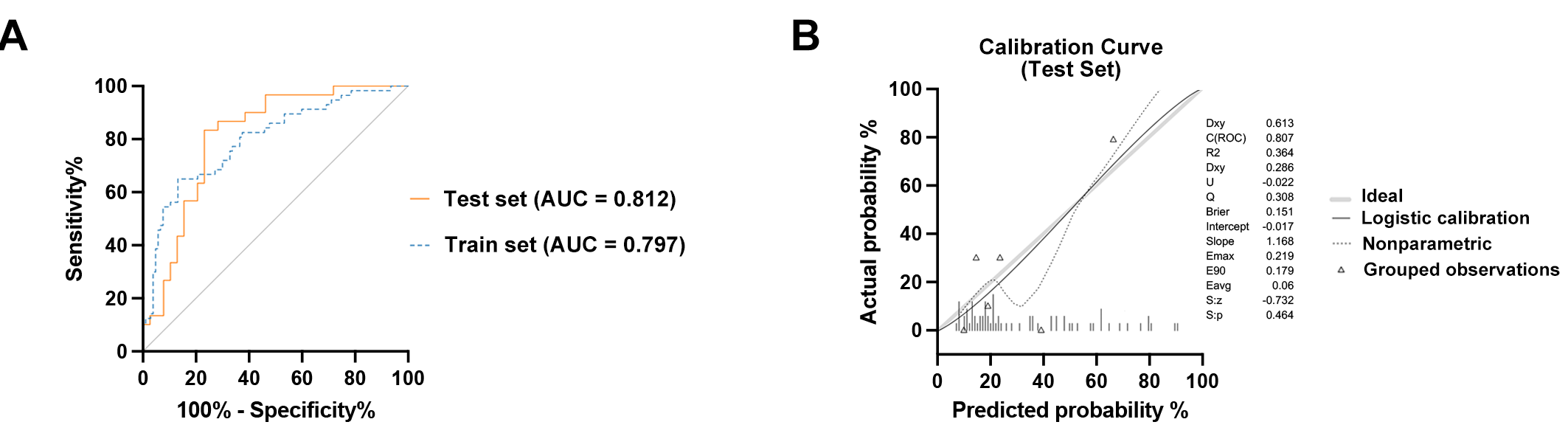
**

**Fig. S2** Model performance assessment in internal split-sample validation. (A) ROC curves from a representative split derived from the repeated validation process. (B) The calibration curve in the testing set showed good agreement between predicted and observed probabilities.

| **Table S1** Significantly altered cerebrospinal fluid metabolites in Parkinson's disease patients compared to Prodromal and Healthy Control groups. | | | |
| --- | --- | --- | --- |
| Metabolites | f.value | p.value | FDR |
| Dopamine 3-o-sulfate | 101.63 | 5.0112e-39 | 1.2428e-36 |
| Cadaverine | 55.405 | 6.5822e-23 | 8.1619e-21 |
| N-Acetylputrescine | 53.466 | 3.4474e-22 | 2.8499e-20 |
| N1-Acetylspermidine | 30.417 | 2.4232e-13 | 1.5024e-11 |
| Putrescine | 20.321 | 2.7873e-09 | 1.3825e-07 |
| 5-Hydroxyindoleacetic acid | 18.084 | 2.299e-08 | 9.5024e-07 |
| Caffeine | 16.446 | 1.087e-07 | 3.8512e-06 |
| Paraxanthine | 15.614 | 2.3993e-07 | 7.4379e-06 |
| Xanthosine | 11.532 | 1.2031e-05 | 0.00030321 |
| (3-O-sulfo)GalCer(d18:1/24:0) | 11.516 | 1.2226e-05 | 0.00030321 |
| HexCer(d18:1/24:0) | 11.284 | 1.5296e-05 | 0.00034485 |
| Spermidine | 11.103 | 1.8208e-05 | 0.00036329 |
| (3-O-sulfo)GalCer(d18:1/24:1) | 11.057 | 1.9043e-05 | 0.00036329 |
| Homoserine | 10.337 | 3.8186e-05 | 0.00067645 |
| HexCer(d18:1/24:1) | 9.9452 | 5.5851e-05 | 0.0009234 |
| Threonine | 9.8248 | 6.2767e-05 | 0.00097289 |
| 1-Methylxanthine | 9.5757 | 7.9929e-05 | 0.001166 |
| Hypoxanthine | 9.513 | 8.495e-05 | 0.0011704 |
| GalCer(d18:1/24:0) | 8.95 | 0.0001468 | 0.0017778 |
| Ergothioneine | 8.9291 | 0.00014982 | 0.0017778 |
| Trimethylamine-N-oxide | 8.9242 | 0.00015054 | 0.0017778 |
| GalCer(d18:1/24:1) | 8.6302 | 0.00020041 | 0.0022592 |
| PE(40:4) | 8.4207 | 0.00024576 | 0.0026057 |
| Nicotimide riboside | 8.3943 | 0.00025217 | 0.0026057 |
| GalCer(d18:1/22:0) | 8.2089 | 0.00030209 | 0.0029968 |
| Acetylcarnitine | 8.0161 | 0.0003646 | 0.0033778 |
| Pyroglutamic acid | 8.0073 | 0.00036775 | 0.0033778 |
| GalCer(d18:1/20:0) | 7.9004 | 0.00040817 | 0.0036152 |
| Propionylcarnitine | 7.8185 | 0.00044213 | 0.003781 |
| Carnitine | 7.7235 | 0.0004851 | 0.0040102 |
| Xanthine | 7.5421 | 0.0005791 | 0.0046328 |
| Uridine | 7.4184 | 0.00065347 | 0.005039 |
| HexCer(d18:1/22:0) | 7.3921 | 0.00067051 | 0.005039 |
| N1-N8-Diacetylspermidine | 7.3039 | 0.00073087 | 0.0053311 |
| SM(d18:1/24:1) | 7.2451 | 0.0007741 | 0.0054379 |
| GalCer(d18:1/18:0) | 7.1973 | 0.00081113 | 0.0054379 |
| Kynurenine | 7.1971 | 0.00081131 | 0.0054379 |
| Phenylalanine | 7.1518 | 0.00084807 | 0.0055348 |
| Homocysteine | 7.0731 | 0.00091587 | 0.0056928 |
| Epinephrine | 7.0667 | 0.00092169 | 0.0056928 |
| Trigonelline | 7.0243 | 0.00096073 | 0.0056928 |
| PE(40:6) | 7.0207 | 0.0009641 | 0.0056928 |
| N-Acetylserine | 6.9718 | 0.0010113 | 0.0058327 |
| N-Alpha-acetyllysine | 6.8612 | 0.001127 | 0.0062248 |
| CE(20:5) | 6.8589 | 0.0011295 | 0.0062248 |
| Cystine | 6.7341 | 0.0012763 | 0.0068808 |
| Glutamic acid | 6.6609 | 0.0013711 | 0.0072347 |
| Hexanoylcarnitine | 6.6043 | 0.0014494 | 0.0074884 |
| Glycine | 6.4445 | 0.0016949 | 0.0085785 |
| HexCer(d18:1/18:0) | 6.3803 | 0.0018051 | 0.0089534 |
| Sarcosine | 6.3199 | 0.0019152 | 0.0091938 |
| Octanoylcarnitine | 6.3133 | 0.0019277 | 0.0091938 |
| Uric acid | 5.963 | 0.0027182 | 0.012719 |
| Lysine | 5.9307 | 0.0028059 | 0.012886 |
| GalCer(d18:2/22:0) | 5.7494 | 0.0033524 | 0.015116 |
| GalCer(d18:1/16:0) | 5.7104 | 0.0034835 | 0.015427 |
| Leucine | 5.6465 | 0.003709 | 0.016137 |
| GlcCer(d18:1/22:0) | 5.5788 | 0.0039644 | 0.016951 |
| GM3(d40:1) | 5.5456 | 0.0040957 | 0.017093 |
| Valerobetaine | 5.5119 | 0.0042339 | 0.017093 |
| PI(18:1/18:1) | 5.5065 | 0.0042561 | 0.017093 |
| Glucose | 5.5025 | 0.0042732 | 0.017093 |
| BMP(22:6/22:6) | 5.3387 | 0.0050199 | 0.019751 |
| GalCer(d18:1/22:1) | 5.3118 | 0.0051544 | 0.019751 |
| Arabitol | 5.3074 | 0.0051766 | 0.019751 |
| PE(36:1) | 5.1797 | 0.0058693 | 0.022054 |
| Asparagine | 5.1497 | 0.0060454 | 0.022377 |
| Creatine | 5.1283 | 0.0061743 | 0.022518 |
| GalCer(d18:2/18:0) | 5.1093 | 0.0062907 | 0.02261 |
| Spermine | 5.0842 | 0.0064481 | 0.022845 |
| Cer(d18:1/24:1) | 5.0613 | 0.0065948 | 0.023035 |
| (3-O-sulfo)GalCer(d18:1/24:0(2OH)) | 5.0096 | 0.0069392 | 0.02364 |
| Adenosine | 5.0068 | 0.0069586 | 0.02364 |
| Choline | 4.9873 | 0.0070935 | 0.023773 |
| Glutamine | 4.9688 | 0.0072237 | 0.023886 |
| Ornithine | 4.8653 | 0.0079986 | 0.026101 |
| Mannose | 4.7282 | 0.0091558 | 0.029489 |
| alpha-GalCer(d18:1/16:0) | 4.6829 | 0.0095736 | 0.030022 |
| Isovalerylcarnitine | 4.6828 | 0.0095741 | 0.030022 |
| PS(18:0_22:6) | 4.6712 | 0.0096845 | 0.030022 |
| 8-Hydroxy-deoxyguanosine | 4.5685 | 0.010717 | 0.032812 |
| GalCer(d18:2/20:0) | 4.536 | 0.011065 | 0.033464 |
| Butyrylcarnitine | 4.401 | 0.012641 | 0.037771 |
| Citrulline | 4.3141 | 0.013772 | 0.040661 |
| Aspartic acid | 4.2899 | 0.014105 | 0.040881 |
| N6,N6,N6-Trimethyllysine | 4.2848 | 0.014176 | 0.040881 |
| Tryptophan | 4.1982 | 0.01544 | 0.044014 |
| CE(22:6) | 4.1621 | 0.016002 | 0.045096 |
| (3-O-sulfo)GalCer(d18:1/16:0) | 4.1125 | 0.016804 | 0.046825 |
| Decanoylcarnitine | 4.0503 | 0.017869 | 0.048999 |
| PE(38:6) | 4.037 | 0.018105 | 0.048999 |
| PE(P-16:0/20:4) | 4.0329 | 0.018177 | 0.048999 |
| TG(18:0_36:2) | 4.0183 | 0.018442 | 0.049178 |
